# Supplementary material for: Study on the Preparation and Effect of Tomato Seedling Disease Biocontrol Compound Seed-Coating Agent
Source: Life (Basel). 2022 Jun 7;12(6):849. doi: 10.3390/life12060849 (PMC9225546; doi:10.3390/life12060849)
Supplement: Supplementary file 1 [file life-12-00849-s001.zip › life-1730759-supplementary.pdf]

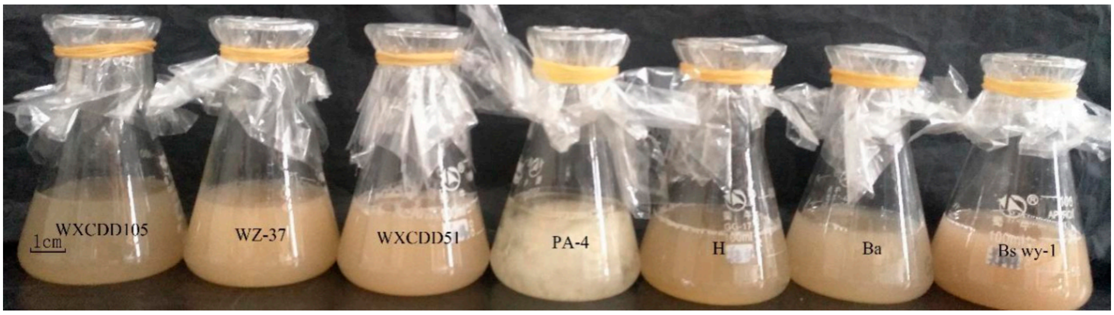

Figure S1. Inhibitory effect of biocontrol bacteria on mycelial growth of *Pythium aphanidermatum*

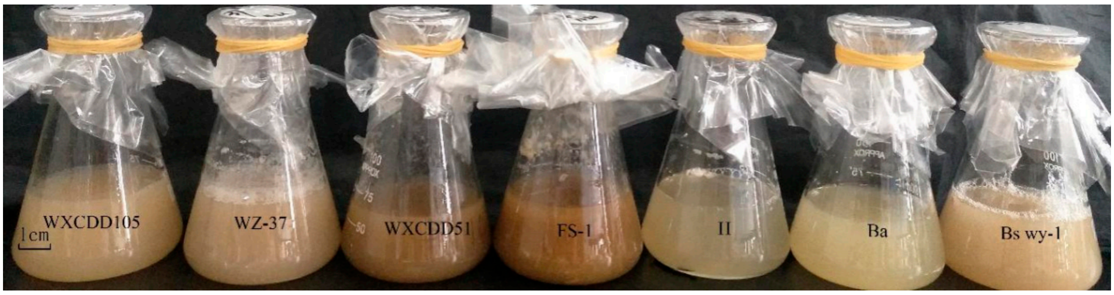

Figure S2. Inhibitory effect of biocontrol bacteria on mycelial growth of *Fusarium sp.*

Table S1. Experimental levels of Five biocontrol bacteria in first orthogonal test

Test number	Factor					Relative germination rate of seeds
	WXCDD51	Ba	WZ-37	WXCDD105	Bs wy-1	
1	1	1	1	1	1	34.4±3.8 bcd
2	1	2	2	2	2	37.8±5.1 bcd
3	1	3	3	3	3	43.3±3.3 b
4	1	4	4	4	4	27.8±1.9 d
5	2	1	2	3	4	26.7±10 d
6	2	2	1	4	3	46.7±3.3 ab
7	2	3	4	1	2	37.8±1.9 bcd
8	2	4	3	2	1	30±8.8 cd
9	3	1	3	4	2	45.6±3.8 ab

10	3	2	4	3	1	55.6±3.8 a
11	3	3	1	2	4	41.1±1.9 bc
12	3	4	2	1	3	36.7±5.8 bcd
13	4	1	4	2	3	36.7±8.8 bcd
14	4	2	3	1	4	43.3±3.3 b
15	4	3	2	4	1	27.8±5.3 d
16	4	4	1	3	2	38.9±5.7 bcd
K1	36.1	34.0	37.2	38.1	37.0	
K2	32.8	43.6	32.0	35.6	38.4	
K3	42.3	36.9	39.1	41.1	38.9	
K4	36.7	33.3	39.5	33.1	33.6	
R	9.5	10.3	7.5	8.1	5.3	

Note：The data in the table shows the mean ± standard deviation, and the lowercase English letters denotes that the difference is significant at the 0.05 level. In the table 1, 2, 3, 4 represent the dilution factor of the original bacterial solution (10^9 cfu/mL), which are 200 times, 300 times, 500 times, and 1000 times respectively. K1, K2 and K3 are the average values of the sum of the corresponding test index values of "1", "2" and "3".R is (range = maximum average yield minimum average yield)

Table S2. Experimental levels of five biocontrol bacteria in second orthogonal test

Test number	Factor					Relative germination rate of seeds
	WXCD D51	Ba	WZ-37	WXCDD105	Bs wy-1	
1	1	1	1	1	1	43.3±3.3bcd
2	1	2	2	2	2	42.2±3.8cd
3	1	3	3	3	3	45. 6±5.1abcd
4	1	4	4	4	4	56. 7±3.3ab
5	2	1	2	3	4	46. 7±10abcd
6	2	2	1	4	3	53. 3±15.2abc

7	2	3	4	1	2	52. 2±1.9abc
8	2	4	3	2	1	57. 8±10.7a
9	3	1	3	4	2	45. 6±3.8abcd
10	3	2	4	3	1	45. 6±1.9abcd
11	3	3	1	2	4	37. 8±6.9d
12	3	4	2	1	3	46. 7±3.3abcd
13	4	1	4	2	3	55. 6±5.1abc
14	4	2	3	1	4	42. 2±1.9cd
15	4	3	2	4	1	56.7±8.8ab
16	4	4	1	3	2	52.2±3.8abc
K1	46.9	47.8	46.6	46.1	50.8	
K2	52.5	45.8	48.1	48.4	48.1	
K3	43.9	48.1	47.8	47.5	50.3	
K4	51.7	53.4	52.5	53.1	45.9	
R	8.6	7.5	5.9	7.0	5.0	

Note: In the table 1, 2, 3, 4 represent the dilution factor of the original bacterial solution (10^9 cfu/mL).1, 2, 3, 4 in WXCDD51 column are 400 times, 500 times, 600 times, 700 times respectively;1, 2, 3, 4 in the Ba column are 250 times, 300 times, 350 times, and 400 times respectively;1, 2, 3, 4 in column WZ-37 are 1100 times, 1000 times, 800 times, and 700 times respectively;1, 2, 3, 4 in column WXCDD105 are 400 times, 500 times, 600 times, and 700 times respectively;1, 2, 3, 4 in the Bs wy-1 column are 400 times, 500 times, 600 times, and 700 times respectively.K1, K2 and K3 are the average values of the sum of the corresponding test index values of "1", "2" and "3".R is (range = maximum average yield minimum average yield)

Table S3. Experimental levels of seed coating in first orthogonal test

Test number	Factor				Relative germination rate of seeds
	Thickener	Film former	Dispersant	Pearlescent pigment	
1	1	1	1	1	92±2 ab
2	1	2	2	2	84.7±6.5 c
3	1	3	3	3	91.3±4.2 ab
4	2	1	2	3	92.7±2.3 ab
5	2	2	3	1	94.7±3.1 a
6	2	3	1	2	86.7±3.1 bc
7	3	1	3	2	75.3±2.3 d
8	3	2	1	3	58.7±2.3 e
9	3	3	2	1	2.7±3.1 f
K1	89.3	86.7	79.1	63.1	
K2	91.4	79.3	60	82.2	

K3	45.6	60.2	87.1	80.9
R	45.8	26.5	27.1	19.1

Note:In the thickener, 1, 2, and 3 respectively represent diatomaceous earth, fumed silica, and kaolin; in the film forming agent, 1, 2, 3, respectively represent gum arabic, polyethylene glycol, and polyvinylpyrrolidone; in the dispersant, 1 , 2, 3 represent sodium lignosulfonate, open powder, sodium dodecylbenzene sulfonate, respectively.K1, K2 and K3 are the average values of the sum of the corresponding test index values of "1", "2" and "3".R is (range = maximum average yield minimum average yield)

Table S4. Experimental levels of seed coating in second orthogonal test

Test number	factor				Relative germination rate of seeds
	Fumed silica	Acacia seyal	SDBS	Pearlescent pigment	
1	1	1	1	1	74.7±1.2 bc
2	1	2	2	2	70±2 c
3	1	3	3	3	74±10 bc
4	2	1	2	3	82.7±3.1 b
5	2	2	3	1	77.3±12.1 bc
6	2	3	1	2	96.7±4.2 a
7	3	1	3	2	76.7±3.1 bc
8	3	2	1	3	97.3±1.2 a
9	3	3	2	1	74±4 bc
K1	72.9	78	89.6	75.33	
K2	85.6	81.5	75.6	81.1	
K3	82.6	81.6	76.0	84.7	
R	12. 7	3.6	14.0	9.3	

Note: 1, 2 and 3 in fumed silica represent 0.5 g, 1 g and 1.5 g; respectively; 1, 2 and 3 in Arabic gum represent 0.5 g, 0.8 g and 1g respectively; 1, 2 and 3 in sodium dodecyl benzene sulfonate represent 0.2 g, 0.5 g and 0.8g respectively; 1, 2 and 3 of pearl powder represent 1.2 g, 1 g and 0.8 g respectively.K1, K2 and K3 are the average values of the sum of the corresponding test index values of "1", "2" and "3".R is (range = maximum average yield minimum average yield).

Table S5. Experimental levels of seed coating in third orthogonal test

Test number	factor				Relative germination rate of seeds
	Fumed silica	Acacia seyal	SDBS	pearlescent pigment	
1	1	1	1	1	94.7±1.2 ab
2	1	2	2	2	94±5.2 ab
3	1	3	3	3	82±3.5 c
4	2	1	2	3	89.3±2.3 b
5	2	2	3	1	82.7±6.1 c
6	2	3	1	2	94.7±2.3 ab
7	3	1	3	2	83.3±3.1 c
8	3	2	1	3	96.7±3.1 a
9	3	3	2	1	94.7±1.2 ab
K1	90.2	89.1	95.3	92.0	
K2	89.2	91.5	92.7	91.3	
K3	91.6	90.4	83	88.7	
R	2.4	2.4	12.3	3.3	

Note: 1, 2 and 3 in fumed silica represent 0.8 g, 1 g and 1.3 respectively; 1, 2 and 3 in Arabic gum represent 0.9 g, 1 g and 1.2g respectively; 1, 2 and 3 in sodium dodecyl benzene sulfonate represent 0.05

g, 0.2 g and 0.3g respectively; 1, 2 and 3 of pearl powder represent 0.5 g, 0.8 g and 0.9 g respectively.K1, K2 and K3 are the average values of the sum of the corresponding test index values of "1", "2" and "3".R is (range = maximum average yield minimum average yield)
